# Cs_2_NaBi_0.6_Er_0.4_Cl_6_ Double-Perovskite Nanoparticles for Hygroscopicity-Assisted Latent Fingerprint Development on Frosted Non-Porous Substrates

**DOI:** 10.3390/nano16110649

**Published:** 2026-05-22

**Authors:** Runkai Hu, Fang Zhou, Yue Zhou, Shangqi Feng, Ziyin Zhang, Yujing Zhao, Li Liu

**Affiliations:** 1 Investigation College, People’s Public Security University of China, Beijing 100038, China; 2 Department of Criminal Science and Technology, Department of Foundation Course, Hunan Police College, Changsha 410138, China; 3 School of Physics and Electronics, Synergetic Innovation Center for Quantum Effects and Application, Key Laboratory of Low-Dimensional Quantum Structures and Quantum Control of Ministry of Education, Key Laboratory for Matter Microstructure and Function of Hunan Province and Institute of Interdisciplinary Studies, Hunan Normal University, Changsha 410081, China; 4 School of Electrnic Information and Physics, Central South University, Changsha 410083, China; 5 School of Physics, Electronictechnology and Intelligent Manufacturing, Huaihua University, Huaihua 418008, China; 6 School of Physics and Electronics, Hunan University, Changsha 410082, China; 7 School of Mathematics, Computer Science and Engineering, University of London, London EC1V 0HB, UK

**Keywords:** Cs_2_NaBi_0.6_Er_0.4_Cl_6_ fluorescent nanopowder, latent fingerprint development, frosted non-porous substrates, forensic science

## Abstract

Latent fingerprint development on rough non-porous substrates using fingerprint powders remains challenging because surface microstructures reduce particle-adhesion selectivity and weaken the contrast between ridges and the background. In this study, Cs_2_NaBi_0.6_Er_0.4_Cl_6_ double-perovskite nanoparticles were prepared by a solvothermal method and investigated as fingerprint-development particles for latent fingerprints on frosted plastic substrates. Structural characterization by X-ray diffraction (XRD), scanning electron microscopy (SEM), Raman spectroscopy, and X-ray photoelectron spectroscopy (XPS) indicated that Er^3+^ was incorporated into the host matrix and that the product consisted of spherical nanoparticles with smooth surfaces, relatively uniform particle-size distribution, and good dispersibility. Comparative experiments involving 40 categories of latent fingerprint samples showed that the Cs_2_NaBi_0.6_Er_0.4_Cl_6_ nanoparticles outperformed conventional powders in developing fingerprints on frosted plastic substrates. Quantitative grayscale analysis using Image J 1.53K and Origin 2024 further showed that the development contrast, expressed as the *D* value, reached 51.21 for sebum-rich fingerprints and 35.87 for oil-contaminated model fingerprints, both of which were higher than those obtained with the other three powders. Because the fluorescence of Cs_2_NaBi_0.6_Er_0.4_Cl_6_ under UV excitation was weaker than that of the commercial red fluorescent powder, we attribute the improved development performance mainly to selective adhesion of the particles to fingerprint residues rather than to fluorescence intensity alone. In addition, the material maintained good performance for aged fingerprints within 10 days and for developed fingerprints stored for up to 8 days. These results suggest that selective residue-affinitive adhesion, possibly assisted by the hydrophilic or moisture-affinitive nature of the ionic double-perovskite particles, plays an important role in improving fingerprint development on rough non-porous substrates. This study provides a physical perspective for latent fingerprint development on rough non-porous substrates and broadens the forensic-science application of lead-free double-perovskite nanomaterials.

## 1. Introduction

Latent fingerprint development is one of the fundamental techniques for personal identification in forensic science. Among existing methods, powder dusting remains one of the most widely used approaches because it is simple, inexpensive, and suitable for on-site application [1]. However, on rough non-porous substrates such as frosted plastics, its effectiveness is often severely limited. Surface microstructures reduce the adhesion selectivity of powder particles and increase background staining [2,3]. As a result, the contrast between fingerprint ridges and the background decreases markedly, making ridge details difficult to recover [3]. To overcome these limitations, techniques such as cyanoacrylate fuming, small-particle reagent treatment, and vacuum metal deposition have been applied to difficult substrates. Nevertheless, these methods often suffer from strong substrate dependence, complex operating procedures, or high equipment cost [4,5]. In comparison, powder dusting remains attractive for practical use, but its effectiveness largely depends on interfacial interactions between powder particles and fingerprint residues [6]. The key issue is therefore to design particles that adhere selectively to ridge residues while suppressing nonspecific retention on rough background regions.

**Figure 1:**
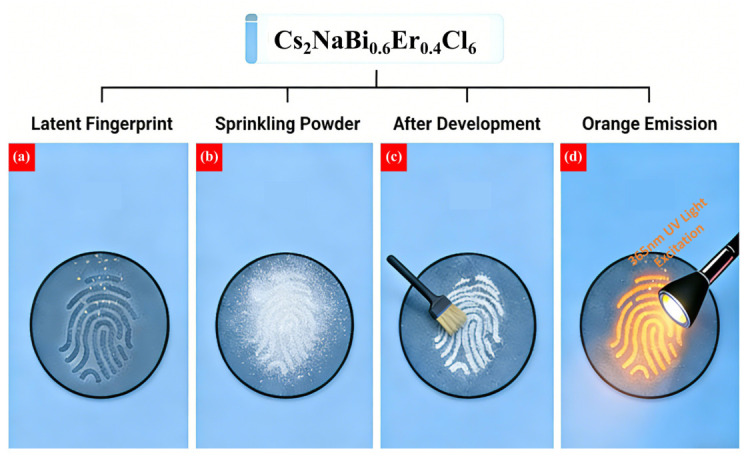
Schematic illustration of the latent fingerprint development process:(**a**) locating the latent fingerprint position; (**b**) sprinkling the fingerprint development powder; (**c**) brushing off excess powder; (**d**) illuminating with a multi-wavelength light source.

From a physical perspective, latent fingerprint development is an interfacial process involving interactions between solid particles and complex biological residues containing water, lipids, and salts. On rough non-porous substrates, the adhesion selectivity of conventional powders usually decreases significantly because surface irregularities promote random particle retention. At the same time, surface undulations hinder the formation of continuous particle adhesion along fingerprint ridges. Raised regions contain more residues and therefore retain more powder, whereas recessed regions contain fewer residues and retain little powder. This eventually leads to discontinuous ridge patterns and a marked decrease in ridge–furrow contrast. Under these conditions, water-assisted capillary interactions may be particularly important [7]. If the powder has sufficient moisture affinity, it can interact more effectively with residue-rich ridge regions, and this interaction can enhance selective adhesion. The latent fingerprint development process is illustrated in Figure 1. First, the fingerprint-development powder is evenly applied to the latent fingerprint (Figure 1a). The powder selectively adheres to and enriches the papillary ridge regions containing sebaceous and sweat residues (Figure 1b) while showing little adhesion to the background regions or furrows. After the excess powder is removed, the fingerprint pattern becomes clearly visible (Figure 1c). Finally, illumination with a multiband light source provides latent fingerprint images with enhanced contrast (Figure 1d).

Environmentally benign lead-free double perovskites provide a useful platform for this type of application [8,9,10,11,12,13,14,15,16,17,18,19,20,21,22,23]. Their ionic-crystal nature gives the materials a certain degree of moisture affinity [2,3,4,5,6], while rare-earth doping can further regulate their optical properties [14,15,16,17,18,19,20]. Among these materials, Cs_2_NaBiCl_6_ is a representative double-perovskite system with favorable optical properties and stability [24]. Doping with metal ions that have an ns^2+^ electronic configuration can improve luminescence-related properties by relaxing parity-forbidden transitions [15,25,26,27,28]. Recent studies and reviews have also highlighted progress in lead-free perovskite materials for optical, catalytic, and bio-related applications, as well as the importance of surface/interface design in improving their stability and functional performance [29,30,31,32,33]. Although moisture affinity is usually regarded as a disadvantage in optoelectronic applications, it may have a positive effect in fingerprint development by promoting particle–residue interactions. Many perovskite-based fingerprint reagents reported in recent years still involve lead-containing components or have mainly been demonstrated on relatively smooth substrates [34]. From a forensic-science perspective, lead-free perovskite powders are preferable because they reduce toxicological risks during routine handling [35,36]. However, the application of lead-free double-perovskite powders to latent fingerprint development on rough non-porous substrates, especially frosted plastics, remains insufficiently investigated [37]. Therefore, this study focuses on the forensic application of Cs_2_NaBi_0.6_Er_0.4_Cl_6_ nanoparticles.

It should be noted that Er^3+^-doped Cs_2_NaBiCl_6_-based double-perovskite materials have previously been reported as downshifting luminescent materials, multifunctional luminescent materials, or temperature-sensing materials [38,39,40,41]. Therefore, the novelty of this study does not lie in the first synthesis of the Cs_2_NaBi_0.6_Er_0.4_Cl_6_ system. Rather, it lies in applying Cs_2_NaBi_0.6_Er_0.4_Cl_6_ nanoparticles with a specific composition to latent fingerprint development on frosted non-porous substrates and in interpreting their development advantage on rough surfaces from the perspective of selective particle–fingerprint residue adhesion. Unlike previous studies, which mainly focused on luminescence properties or optical applications, this study evaluates the suitability, contrast advantage, and aged-fingerprint development capability of this powder in forensic latent fingerprint development. Cs_2_NaBi_0.6_Er_0.4_Cl_6_ nanoparticles were successfully synthesized by a solvothermal method and used in powder form to develop latent fingerprints on frosted plastic substrates. By combining structural characterization, qualitative observation, and quantitative grayscale analysis, this study found that the material exhibited good sensitivity, high contrast, and a certain photoluminescence response, making it particularly suitable for addressing the difficulty of latent fingerprint development on frosted plastic surfaces. These results reveal the physical characteristics of particle–residue interactions on rough non-porous surfaces and provide an effective strategy for designing functional fingerprint development materials.

## 2. Experimental Section

### 2.1. Preparation of Cs_2_NaBi_0.6_Er_0.4_Cl_6_ Nanoparticles

All reagents used in the experiments were of analytical grade and were used without further purification. The chemical reagents included cesium chloride (CsCl, 99.9%, Innochem, Beijing, China), sodium chloride (NaCl, 99.99%, Shanghai Aladdin Biochemical Technology, Shanghai, China), bismuth chloride (BiCl_3_, 99.99%, Yuanye Biotechnology, Shanghai, China), erbium chloride (ErCl_3_, 99.9%, Rhawn Reagents, Shanghai, China), concentrated hydrochloric acid (HCl, 35.0%, guaranteed reagent grade), and reverse-osmosis bottled water (Hangzhou Wahaha Group, Hangzhou, China). All chemicals were used as received. The experimental materials included gold-colored powder (Cu–Zn alloy), silver-colored powder (Al-based powder), red fluorescent powder, and Jinlongyu vegetable oil.

Cs_2_NaBi_0.6_Er_0.4_Cl_6_ nanoparticles were synthesized via a solvothermal method, as follows. In a 100.0 mL polytetrafluoroethylene (PTFE) liner, 7.69 g (45.68 mmol) of CsCl, 1.33 g (22.84 mmol) of NaCl, 2.50 g (9.14 mmol) of ErCl_3_, and 4.32 g (13.71 mmol) of BiCl_3_ were dissolved in 80.00 mL of HCl. The precursor solution was transferred to a high-pressure autoclave and heated at 180 °C for 12 h. After naturally cooling to room temperature, the resulting powder was washed twice with isopropanol and then dried in an oven at 60 °C for 12 h. The dried Cs_2_NaBi_0.6_Er_0.4_Cl_6_ powder was stored in a sealed sample vial in a desiccator until use. Apart from dry sealed storage, no additional coating or surface-passivation treatment was applied to the powder in this study.

### 2.2. Sample Characterization

The microstructure of Cs_2_NaBi_0.6_Er_0.4_Cl_6_ was observed by scanning electron microscopy (NovaSEM 450, FEI Company, Hillsboro, OR, USA). The crystal structure was analyzed using an X-ray diffractometer (D8 DISCOVER, Bruker AXS GmbH, Karlsruhe, Germany). X-ray photoelectron spectroscopy (ESCALAB 250Xi, Thermo Fisher Scientific, East Grinstead, UK) was used to identify the elemental composition and chemical states of the prepared material. Raman spectroscopy (LabRAM HR, HORIBA France SAS, Villeneuve-d’Ascq, France) was used to investigate photon–molecule or photon–atom interactions in Er^3+^-doped Cs_2_NaBiCl_6_ and to analyze the relationship between its structure and its properties. Photoluminescence (PL) spectra, photoluminescence excitation (PLE) spectra and time-resolved photoluminescence (TRPL) decay curves were measured using an Edinburgh FLs1000 spectrometer (Edinburgh Instruments Ltd., Livingston, UK).

### 2.3. Latent Fingerprint Development

All latent fingerprint samples were provided by one volunteer donor with informed consent. At least five independent samples were prepared for each powder, substrate, and fingerprint type. Natural-residue fingerprints were prepared as follows. The donor washed his/her hands with soap, allowed them to dry naturally, and then wore disposable gloves for 10 min to promote residue accumulation. Fingerprints were subsequently deposited on the substrates with a contact time of 2 s, during which the applied pressure was kept as constant as possible. Oil-contaminated model fingerprints were prepared by applying a small and controlled amount of vegetable oil to the fingertip before deposition. These samples were used only to simulate contact with exogenous oil contamination and should not be regarded as natural oil-contaminated latent fingerprints.

During development, a small quantity of Cs_2_NaBi_0.6_Er_0.4_Cl_6_ nanoparticles was picked up with a fingerprint brush and gently brushed over the fingerprint area on the substrate surface. When ridge details began to appear, the brush was tapped lightly to remove excess powder. Brushing was then continued along the ridge-flow direction until clear details were obtained. All other samples were treated using the same procedure. The substrates and fingerprint development powders used in this study are listed in Table 1. Developed fingerprint samples were observed, photographed, and recorded under a multiband light source, after which they were lifted and preserved using fingerprint tape. A unified coding rule was used: powder type was recorded as A for gold-colored powder, B for silver-colored powder, C for red fluorescent powder, or D for Cs_2_NaBi_0.6_Er_0.4_Cl_6_ nanoparticles; substrate type was recorded as 1 for a glass slide, 2 for a stainless-steel cup, 3 for a compact disc, 4 for a smooth plastic cup, and 5 for a frosted plastic cup. For powders C and D, the illumination marker S for white light or U for 365 nm UV light was appended to the sample code.

### 2.4. Quantitative Method for Fingerprint Development Contrast

#### 2.4.1. Image Processing and Analysis

Color images were converted into grayscale images using Adobe Photoshop 2025. When the grayscale value of the papillary ridges was lower than that of the furrows, the grayscale image was inverted. The oriented fingerprint image was then imported into Image J 1.53K. A straight line crossing both papillary ridges and furrows was drawn using the Straight Line tool, with its direction kept as perpendicular as possible to the ridge flow. The line was required to cross more than 10 ridges and furrows. The Plot Profile tool under the Analyze menu was then used to obtain the grayscale profile along the line. In the profile, peaks corresponded to papillary ridges and valleys corresponded to furrows [29].

#### 2.4.2. Software Calculation

The grayscale profile was imported into Origin 2024. On the basis of the fingerprint image and the corresponding grayscale profile, the peaks and valleys were individually identified. The Integration tool was then employed to calculate the area under each peak and each valley. The average intensity was derived from the ratio of this area to the length of the integration interval [30].

#### 2.4.3. Qualitative Analysis

**Figure 2:**
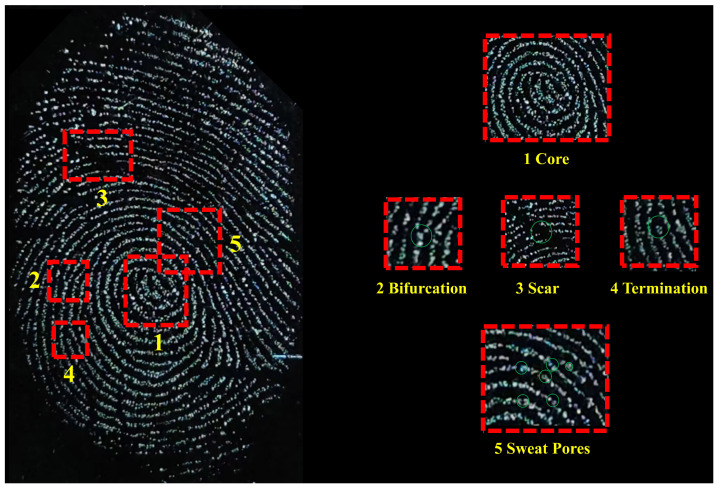
Schematic diagram of fingerprint features at different levels.

To evaluate the fingerprint-development performance of the Cs_2_NaBi_0.6_Er_0.4_Cl_6_ nanoparticles, four different development powders were selected. A total of 40 categories of natural-residue and oil-contaminated model latent fingerprint samples were prepared on five substrate types, and a systematic qualitative analysis was performed. First, the recovery of fingerprint characteristics was comprehensively evaluated. First-level features, second-level features, and some third-level features were assessed according to the hierarchy of forensic fingerprint characteristics. In this study, the criterion of more than eight minutiae, or second-level features, was used as a practical benchmark to evaluate the ability of each powder to recover fingerprint characteristics, as shown in Figure 2.

#### 2.4.4. Quantitative Analysis and Equations

To further investigate the development effect of the Cs_2_NaBi_0.6_Er_0.4_Cl_6_ nanoparticles on latent fingerprints on frosted non-porous substrates, a quantitative analysis method based on development-contrast calculation was introduced. The procedure was as follows. First, a straight line crossing papillary ridges and furrows was selected in the fingerprint image, and the grayscale profile along this line was extracted. The profile variation was then used to determine whether the latent fingerprints developed by different powders clearly presented the corresponding ridge structure. Finally, fingerprint development contrast was quantitatively calculated using the relevant equations.

Three contrast definitions were used in this study [42,43]. In the first definition, the contrast is expressed as the intensity difference between furrows and ridges (*D*):

(1) $$\begin{array}{c} D=\left|I_{v}-I_{r}\right| \end{array}$$

where $I_{v}$ and $I_{r}$ denote the average pixel intensities of the furrow region and ridge region, respectively. In the second definition, the contrast is expressed as the intensity ratio between furrows and ridges (*R*):

(2) $$\begin{array}{c} R=\left|I_{v}/I_{r}\right| \end{array}$$

The third definition uses the Michelson contrast ($C_{m}$), which was proposed for patterns with similar proportions of bright and dark elements:

(3) $$\begin{array}{c} C_{m}=\left|I_{v}-I_{r}\right|/\left|I_{v}+I_{r}\right| \end{array}$$

Here, $I_{r}$ denotes the average intensity of the peaks, and $I_{v}$ denotes the average intensity of the valleys. The average intensity is defined as the integrated area under a segment of the grayscale curve divided by the length of that segment. For each condition, at least five independent fingerprint samples were analyzed. For each image, one line profile crossing more than ten ridge–furrow pairs was extracted. Data are presented as “mean|relative standard deviation” unless otherwise stated.

## 3. Results and Discussion

### 3.1. Morphology, Chemical Composition, and Optical Properties of Cs_2_NaBi_0.6_Er_0.4_Cl_6_ Nanoparticle Powder

**Figure 3:**
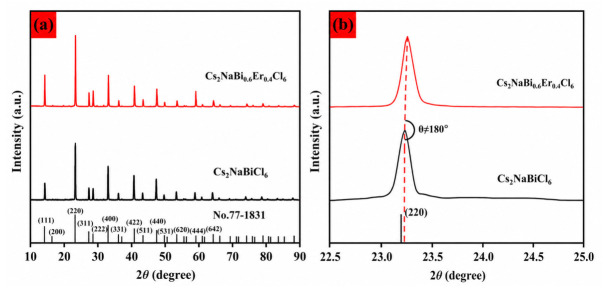
(**a**) XRD patterns of the Cs_2_NaBi_0.6_Er_0.4_Cl_6_ sample. (**b**) Magnified view of the (220) diffraction peak.

The X-ray diffraction (XRD) pattern of the Er^3+^-doped Cs_2_NaBiCl_6_ double perovskite nanocrystals is shown in Figure 3. Figure 3a indicates that the diffraction peaks of the as-prepared Cs_2_NaBi_0.6_Er_0.4_Cl_6_ sample can all be indexed to a cubic structure (space group Fm$\bar{3}$m), consistent with JCPDS card No. 77-1831, confirming that the sample formed the expected double-perovskite main phase. No additional diffraction peaks attributable to secondary phases or Er-doping-related phases were observed. A magnified analysis of the (220) diffraction peak (Figure 3b) reveals that upon Er^3+^ addition, the diffraction peaks gradually shift to higher angles, which may be related to the smaller ionic radius of Er^3+^ compared with the effective radius of the substituted site, causing local lattice contraction. The slight deviation of this peak from the standard card is mainly attributed to instrumental error or differences in synthesis conditions. These findings are broadly consistent with existing reports on Er^3+^-doped Cs_2_NaBiCl_6_ systems [22].

**Figure 4:**
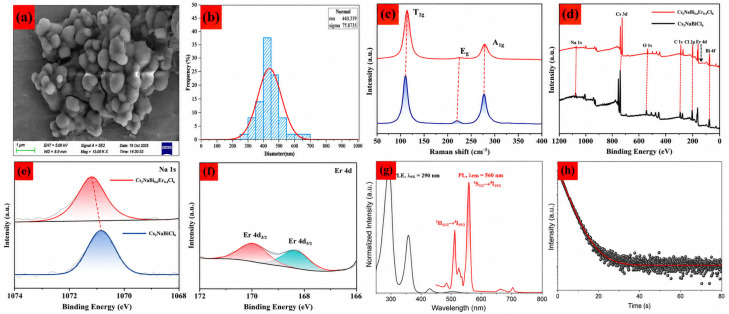
(**a**) SEM image. (**b**) Particle size distribution histogram. (**c**) Raman spectra. (**d**) XPS survey spectra. (**e**) High−resolution Na 1s spectra. (**f**) High−resolution Er 4d spectrum. (**g**) Photoluminescence excitation (PLE) and emission (PL) spectra of Cs_2_NaBi_0.6_Er_0.4_Cl_6_. The black curve is the PLE spectrum acquired by monitoring the emission at 290 nm, and the red curve is the PL emission spectrum obtained under excitation at 560 nm. (**h**) TRPL decay curve of Cs_2_NaBi_0.6_Er_0.4_Cl_6_ nanoparticles.

Figure 4a shows a scanning electron microscopy (SEM) image of Cs_2_NaBi_0.6_Er_0.4_Cl_6_ nanocrystals. The particles are generally quasi-spherical with relatively smooth surfaces and good dispersibility, providing a favorable morphological basis for use as a latent fingerprint development powder. Figure 4b presents a histogram of the particle size distribution histogram; the statistical average particle size is 510.0 nm, a size that is conducive to maintaining a certain degree of flowability while ensuring adequate adsorption performance toward fingerprint residues.

To further investigate the local structure, Raman spectra of Cs_2_NaBi_0.6_Er_0.4_Cl_6_ were recorded at room temperature in the range of 50–400 cm^−1^ to probe the vibrational characteristics of the [BiCl_6_]^3−^ octahedra; the results are shown in Figure 4c. The spectra display three prominent bands associated with the [BiCl_6_]^3−^ octahedra: the low-frequency peak at 108.7 cm^−1^ can be assigned to the Bi-0Cl bending vibration in the $T_{2g}$ mode, corresponding to symmetric bending of the octahedron along one of its axes; the high-frequency peaks at 220.2 and 274.4 cm^−1^ correspond to stretching vibrations in the $E_{g}$ and $A_{1g}$ modes, respectively. The $E_{g}$ mode corresponds to the doubly degenerate bending vibration of the octahedron, which involves pairwise motion of only four chlorine atoms [16,44,45,46]. Compared with those of undoped Cs_2_NaBiCl_6_, the Raman peaks of Cs_2_NaBi_0.6_Er_0.4_Cl_6_ are generally shifted slightly toward higher wavenumbers, indicating that the introduction of Er^3+^ disturbs the local octahedral environment and may alter the average force constant of the metal–chlorine bonds [47,48,49,50].

Furthermore, X-ray photoelectron spectroscopy (XPS) was employed to analyze the valence states and elemental distribution of Cs_2_NaBiCl_6_ and Cs_2_NaBi_0.6_Er_0.4_Cl_6_. Figure 4d presents the survey XPS spectra of both samples: in the undoped sample, signals of Cs 3d, Na 1s, Bi 4f, and Cl 2p are detected; in the Er^3+^-doped sample, an additional Er 4d peak is observed [51,52,53]. The C 1s peak at 285.1 eV was used as a reference for charge correction. Figure 4e shows the high-resolution Na 1s spectra; the Na 1s peaks of Cs_2_NaBiCl_6_ and Cs_2_NaBi_0.6_Er_0.4_Cl_6_ are located at 1070.95 eV and 1071.11 eV, respectively, confirming that Na exists in the +1 oxidation state [53,54,55]. Figure 4f displays the high-resolution Er 4d spectrum of the Er^3+^-doped sample; two peaks appear at 169.87 eV and 168.43 eV, assigned to Er ^4^d_3\2_ and Er ^4^d_5\2_, respectively, further confirming the successful incorporation of Er^3+^. Notably, although no obvious changes are observed in the survey spectra, the high-resolution spectra reveal that upon Er^3+^ doping, the binding energies of elements such as Na are slightly shifted to higher values [56,57,58]. This may be because the substitution of Na^+^ and Bi^3+^ by Er^3+^ causes contraction of the [NaCl_6_]^5−^ and [BiCl_6_]^3−^ octahedra, leading to a change in the electron density of the surrounding elements [12,47].

The visible emission of Cs_2_NaBi_0.6_Er_0.4_Cl_6_ under UV excitation facilitates distinguishing the powder signal from complex backgrounds, thereby enhancing ridge–background contrast during latent fingerprint development. The photoluminescence excitation and emission spectra are shown in Figure 4g. The excitation spectrum was recorded by monitoring the emission at 560 nm, whereas the emission spectrum was obtained under 290 nm excitation. A UV excitation band and a broad visible emission band centered at approximately 560 nm confirm the UV-excited luminescence of Cs_2_NaBi_0.6_Er_0.4_Cl_6_. The sample shows a broad orange-yellow emission band from approximately 500 to 600 nm, which originates from the ^4^H_11\2_→^4^I_15\2_ transition and the ^4^S_3\2_→^4^I_15\2_ transition of Er^3+^ in an octahedral coordination environment. This result is in close agreement with the experimental observations of Xu et al. [14]. Furthermore, the TRPL decay curve monitored at 520 nm was fitted using a tri-exponential function, as shown in Figure 4h. The fitted lifetimes are $\tau_{1}$ = 203.49 μs, $\tau_{2}$ = 2004.70 μs, and $\tau_{3}$ = 4037.38 μs, with an average lifetime of 2080.68 μs (Table 2). The multi-exponential decay may originate from different local environments of Er^3+^ ions and multiple relaxation pathways in the host lattice [59,60,61].

### 3.2. Application of Cs_2_NaBi_0.6_Er_0.4_Cl_6_ Nanoparticles to Latent Fingerprint Development

To evaluate the applicability of the prepared Cs_2_NaBi_0.6_Er_0.4_Cl_6_ nanoparticles in latent fingerprint development, comparative experiments were carried out using gold-colored powder, silver-colored powder, and commercial red fluorescent powder as reference materials. The tested fingerprints included natural-residue fingerprints and oil-contaminated model fingerprints. The comparison focused on ridge detail recovery, ridge–furrow contrast, substrate adaptability, and fluorescence-assisted visualization performance [48,49]. The results showed that the prepared powder had satisfactory fingerprint development capability on various substrates, with the most pronounced advantage on rough non-porous substrates such as frosted plastics. On such surfaces, conventional powders are more likely to produce contrast loss due to roughness-enhanced background retention, whereas the Cs_2_NaBi_0.6_Er_0.4_Cl_6_ nanoparticles maintained better selective retention on fingerprint ridges [48,49].

#### 3.2.1. Qualitative Analysis of Fingerprint Development

**Figure 5:**
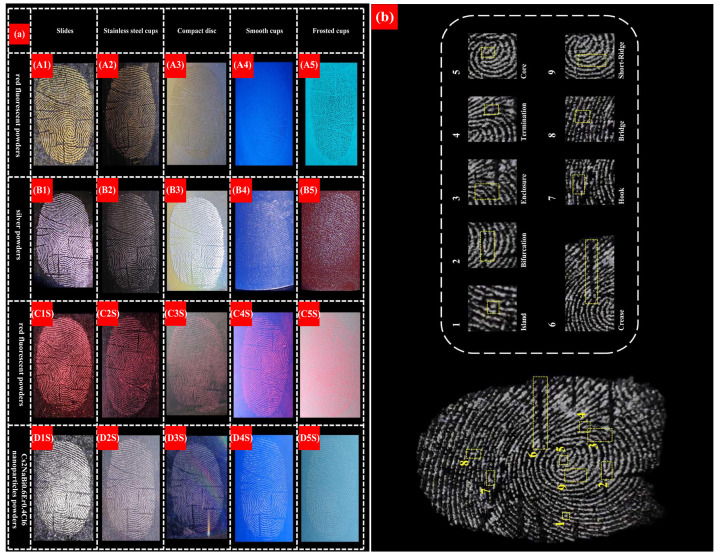
(**a**) Natural-residue latent fingerprints under-white light illumination: gold-colored and silver-colored powders (A1)–(B5); red fluorescent powders and Cs_2_NaBi_0.6_Er_0.4_Cl_6_ nanoparticles powders (C1S)–(D5S); (**b**) Detailed characteristics of fingerprint sample D1.

For sebum-rich fingerprints on different substrates, white-light observation showed that gold-colored powder, silver-colored powder, red fluorescent powder, and Cs_2_NaBi_0.6_Er_0.4_Cl_6_ nanoparticles all exhibited strong ability to recover latent fingerprint morphology. The developed images obtained on five substrates, namely a glass slide, a stainless-steel cup, a compact disc, a frosted plastic cup, and a smooth plastic cup, are shown as A1 to D5S in Figure 5a. Although the latent fingerprints that were developed with gold-colored powder, silver-colored powder, and red fluorescent powder on the frosted plastic cup could still provide identifiable ridge information, several deficiencies remained. As shown in A5 and B5 in Figure 5a, the papillary ridges that were developed with gold-colored and silver-colored powders were frequently interrupted. This was attributed to the rough surface of the frosted plastic cup, which reduced powder adhesion. In the latent fingerprint that was developed with red fluorescent powders (C5S in Figure 5a), a large amount of powder was deposited in the furrows, resulting in a blurred overall image. By contrast, the latent fingerprint that was developed with Cs_2_NaBi_0.6_Er_0.4_Cl_6_ nanoparticles powders (D5S in Figure 5a) showed uniform powder attachment on papillary ridges, continuous and complete ridges, and very little powder accumulation in the furrows. Clear first-level and second-level features, as well as a small number of visible third-level features, could be observed. These observations indicate that Cs_2_NaBi_0.6_Er_0.4_Cl_6_ nanoparticles outperform conventional powders in recovering fingerprint details on frosted plastic materials and can help address the limitations of conventional powder dusting for latent fingerprint development on frosted non-porous substrates in forensic science.

**Figure 6:**
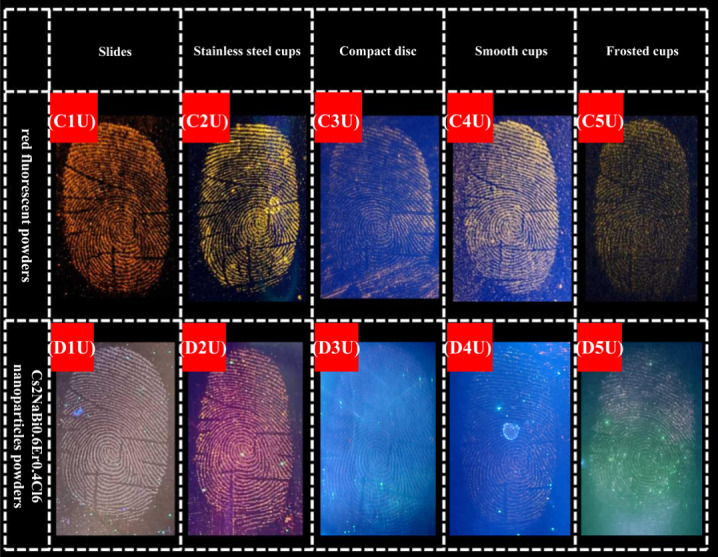
Natural-residue latent fingerprints under 365 nm UV illumination: red fluorescent powders (C1U)–(C5U) and Cs_2_NaBi_0.6_Er_0.4_Cl_6_ nanoparticles powders (D1U)–(D5U).

Gold-colored and silver-colored powders are non-fluorescent. Natural-residue latent fingerprint samples developed with red fluorescent powders and Cs_2_NaBi_0.6_Er_0.4_Cl_6_ nanoparticles powders were observed under 365 nm UV light in a dark-field environment, and the photographs are shown in Figure 6. Both fingerprint development powders produced satisfactory results on glass slides, stainless-steel cups, and smooth plastic cups, allowing sufficiently clear details to be visualized. However, comparison of C3U and D3U in Figure 6 shows that under 365 nm UV illumination, the fluorescence-assisted contrast of fingerprints developed with Cs_2_NaBi_0.6_Er_0.4_Cl_6_ nanoparticles was lower than that obtained with the commercial red fluorescent powders. This result indicates that the prepared powder does not possess a fluorescence advantage in the conventional optical sense. Nevertheless, when combined with the white-light observations on the frosted substrate, these results show that fluorescence intensity is not the dominant factor controlling visualization quality. Instead, the superior performance of the prepared powder on frosted non-porous surfaces mainly originates from its stronger selective adhesion to fingerprint residues.

**Figure 7:**
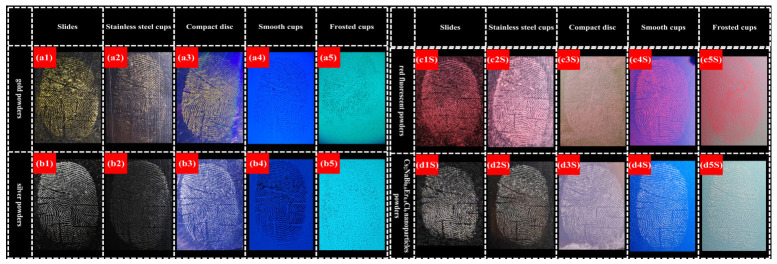
Oil-contaminated model latent fingerprints under white-light illumination: gold-colored and silver-colored powders (a1)–(b5); red fluorescent powders and Cs_2_NaBi_0.6_Er_0.4_Cl_6_ nanoparticles powders (c1S)–(d5S).

Oil-contaminated model fingerprints are also relevant in forensic practice because surfaces may be touched after contact with exogenous oily substances. Under multiband light-source illumination, gold-colored powder, silver-colored powder, red fluorescent powder, and Cs_2_NaBi_0.6_Er_0.4_Cl_6_ nanoparticles all recovered the morphological features of latent fingerprints. The developed images obtained on five substrates are shown from a1 to d5S in Figure 7. The presence of exogenous vegetable oil increased the amount and viscosity of residues in the fingerprint area, which enhanced powder retention. Consequently, the powder more readily accumulated in the furrows of oil-contaminated model fingerprints, blurring features at different levels and reducing the overall development contrast. On frosted non-porous substrates such as frosted plastic cups, the latent fingerprint that was developed with silver-colored powder (b5 in Figure 7) showed frequent interruptions in the papillary ridges. In comparison, the oil-contaminated model latent fingerprint that was developed with gold-colored powder (a5 in Figure 7) was relatively clear, which may be related to enhanced powder retention on the rough surface due to the viscous exogenous oil residues. The latent fingerprint that was developed with red fluorescent powder (c5S in Figure 7) showed substantial powder deposition in the furrows, obscuring detailed features. In contrast, although the ridges that were developed with Cs_2_NaBi_0.6_Er_0.4_Cl_6_ nanoparticles (d5S in Figure 7) became slightly broader because of increased powder attachment, the overall image still retained high contrast and observable ridge details. This performance effectively overcame the limitations of gold-colored powder, silver-colored powder, and red fluorescent powder in developing oil-contaminated model latent fingerprints on frosted non-porous substrates.

**Figure 8:**
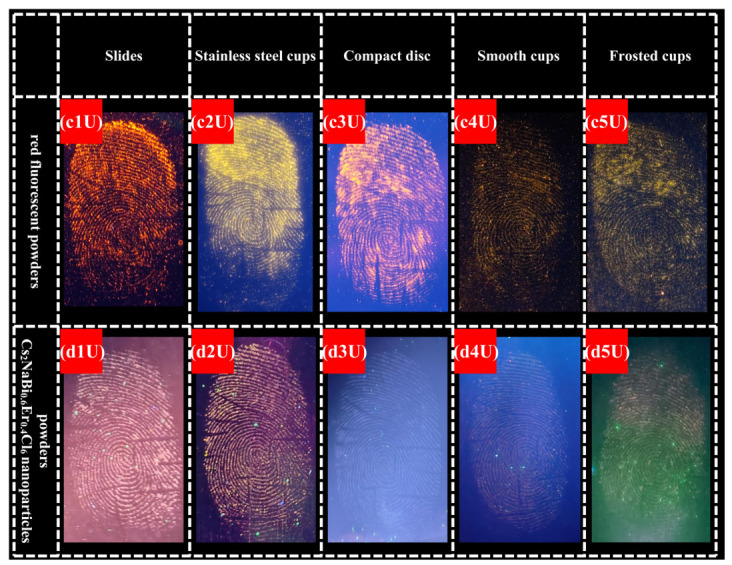
Oil-contaminated model latent fingerprints under 365 nm UV illumination: red fluorescent powders (c1U)–(c5U) and Cs_2_NaBi_0.6_Er_0.4_Cl_6_ nanoparticles powders (d1U)–(d5U).

Oil-contaminated model latent fingerprint samples that had been developed with red fluorescent powder and Cs_2_NaBi_0.6_Er_0.4_Cl_6_ nanoparticles were observed under 365 nm UV light in a dark-field environment, as shown from c1U to d5U in Figure 8. For samples developed with red fluorescent powder, a large amount of powder adhered to the furrows, resulting in blurred overall images. Although first-level features were relatively clear, second-level and third-level features were not obvious, which was unfavorable for forensic fingerprint examination. Cs_2_NaBi_0.6_Er_0.4_Cl_6_ nanoparticles attached more uniformly to papillary ridges and showed less deposition in the furrows. Under 365 nm UV illumination, Cs_2_NaBi_0.6_Er_0.4_Cl_6_ nanoparticles still exhibited relatively weak fluorescence and were affected by background interference, leading to limited fluorescence-assisted contrast in oil-contaminated model latent fingerprint imaging. However, compared with the commercial red fluorescent powder, the prepared powder showed less nonspecific deposition in furrow regions and more uniform retention in ridge regions. These observations further indicate that the final visualization quality on rough non-porous substrates depends more on selective particle adhesion than on fluorescence intensity alone.

#### 3.2.2. Quantitative Analysis of Development Contrast for Latent Fingerprints on Frosted Non-Porous Substrates

**Figure 9:**
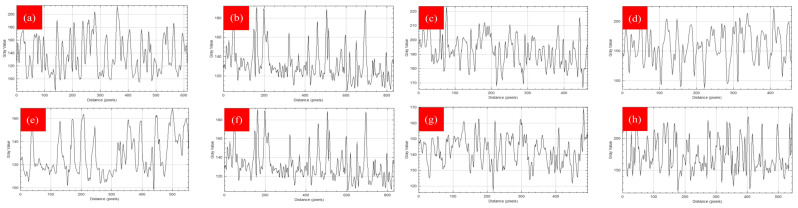
Grayscale graphs of latent fingerprints developed with different powders: (**a**) natural-residue latent fingerprint developed with gold-colored powder; (**b**) natural-residue latent fingerprint developed with silver-colored powder; (**c**) natural-residue latent fingerprint developed with red fluorescent powder; (**d**) natural-residue latent fingerprint developed with Cs_2_NaBi_0.6_Er_0.4_Cl_6_ nanoparticles; (**e**) oil-contaminated model latent fingerprint developed with gold-colored powder; (**f**) oil-contaminated model latent fingerprint developed with silver-colored powder; (**g**) oil-contaminated model latent fingerprint developed with red fluorescent powder; (**h**) oil-contaminated model latent fingerprint developed with Cs_2_NaBi_0.6_Er_0.4_Cl_6_ nanoparticles.

To quantitatively evaluate the latent fingerprint development ability of Cs_2_NaBi_0.6_Er_0.4_Cl_6_ nanoparticles on frosted non-porous substrates, the development contrasts obtained with different powders on frosted plastic substrates, shown in Figure 9a–h, were quantitatively analyzed and compared. Images of fingerprints developed on frosted plastic cups were converted into black-and-white grayscale images using Adobe Photoshop. When the grayscale value corresponding to the papillary ridges was lower than that of the furrows, the grayscale images were inverted. Selective adhesion of powder between papillary ridges and furrows directly caused significant grayscale differences in the images. The ridges with adherent powder showed higher grayscale values and appeared bright white, whereas the furrows without powder adhesion retained the original substrate color and had lower grayscale values, thereby producing clear contrast. Considering the differences in the development performance of different powders on frosted plastic cups, a common feature path present in all fingerprint images was selected for grayscale-value analysis to achieve standardized cross-sample comparison and quantitative calculation.

**Figure 10:**
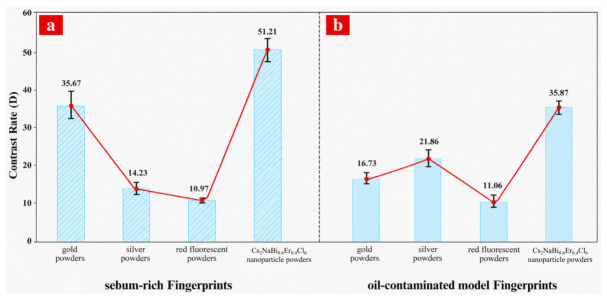
(**a**) Intensity difference (*D*) between furrows and ridges for natural-residue latent fingerprints developed on frosted plastic substrates; (**b**) intensity difference (*D*) between furrows and ridges for oil-contaminated model latent fingerprints developed on frosted plastic substrates.

This study systematically evaluated grayscale images of natural-residue fingerprints (Figure 9a) and oil-contaminated model fingerprints (Figure 9b) developed with four powders on frosted plastic substrates. Three contrast definitions were calculated for each image, namely, the intensity difference between furrows and ridges (*D*), the intensity ratio between furrows and ridges (*R*), and the Michelson contrast ($C_{m}$). The results support the use of the *D*-value definition over the other forms (Table 3), consistent with the findings of Matuszewski et al. [41]. For sebum-rich fingerprints, the contrast (*D* value) obtained with Cs_2_NaBi_0.6_Er_0.4_Cl_6_ nanoparticles reached 51.21, exceeding the values obtained with gold-colored powder, silver-colored powder, and red fluorescent powder, which were 35.67, 14.23, and 10.97, respectively. For oil-contaminated model fingerprints, the contrast reached 35.87, also higher than those obtained with the other three powders, namely, 16.73, 21.86, and 11.06. As shown in Figure 9 and Figure 10, the quantitative data consistently demonstrate that Cs_2_NaBi_0.6_Er_0.4_Cl_6_ nanoparticles provided better latent fingerprint development on frosted plastic surfaces. It is worth noting that the advantage of the prepared powder was most obvious on frosted non-porous substrates. On such substrates, conventional powders lose contrast because of roughness-enhanced background retention, whereas this material retained higher ridge–furrow discrimination, indicating that its performance advantage is closely related to complex rough interfaces rather than merely to smooth substrates.

It should be emphasized that the superior performance of Cs_2_NaBi_0.6_Er_0.4_Cl_6_ nanoparticles on frosted plastic substrates cannot be simply attributed to fluorescence enhancement. In fact, under UV excitation, the fluorescence intensity of the prepared powder was weaker than that of the commercial red fluorescent powder. Nevertheless, the prepared powder still produced higher overall development contrast on frosted substrates. This result indicates that on rough non-porous surfaces, the dominant factor controlling fingerprint visualization is selective adhesion assisted by moisture affinity rather than fluorescence intensity alone. In other words, compared with fluorescence intensity, moisture-affinity-assisted adhesion plays a more important role in determining development contrast on rough non-porous substrates.

#### 3.2.3. Contrast Analysis of Fingerprints Developed with Cs_2_NaBi_0.6_Er_0.4_Cl_6_ Nanoparticles After Different Storage Times

**Figure 11:**
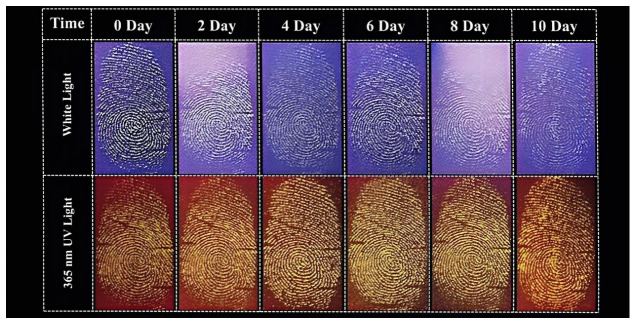
Representative images of latent fingerprints with different aging times developed by Cs_2_NaBi_0.6_Er_0.4_Cl_6_ nanoparticles.

**Figure 12:**
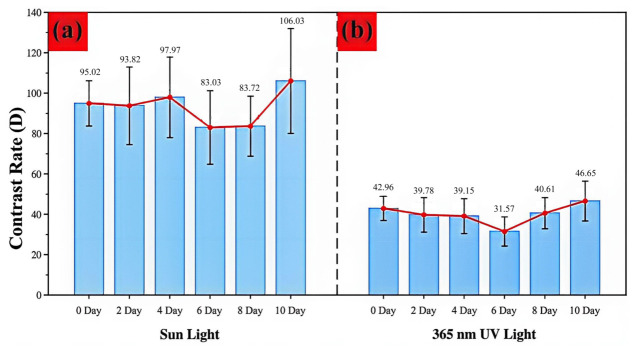
(**a**) Contrast (*D*) of aged fingerprints developed with Cs_2_NaBi_0.6_Er_0.4_Cl_6_ nanoparticle material under natural illumination; (**b**) contrast (*D*) of aged fingerprints developed with Cs_2_NaBi_0.6_Er_0.4_Cl_6_ nanoparticle material under 365 nm ultraviolet (UV) illumination.

To evaluate the ability of Cs_2_NaBi_0.6_Er_0.4_Cl_6_ nanoparticles to develop aged latent fingerprints, latent fingerprint samples from the same finger with different aging times (0, 2, 4, 6, 8, and 10 days) were prepared on frosted plastic surfaces. After the aging period, the samples were developed by dusting, and the development effectiveness was assessed through a combination of qualitative observation and quantitative contrast analysis; the results are presented in Figure 11 and Figure 12.

As shown in Figure 11, as the aging time increases, the latent fingerprint residue gradually undergoes evaporation and degradation, yet Cs_2_NaBi_0.6_Er_0.4_Cl_6_ nanoparticles can still yield readable ridge patterns at 10 days. Compared with fresh fingerprints, some aged samples show slightly reduced ridge width and continuity, but the overall ridge details remain identifiable. The quantitative results in Figure 12 further indicate that under both white light and 365 nm UV light, the contrast *D* remains within an acceptable range over the entire aging period and shows no obvious systematic deterioration. Specifically, under natural light, the contrast *D* of the fresh (0-day) fingerprint is 95.02, while that of the 8-day aged fingerprint still reaches 83.72; under 365 nm UV light, the performance is also satisfactory, with *D* decreasing from 42.96 (0 day) to 40.61 (8 day) in Table 4. It is worth noting that although the fingerprint residue gradually diminishes with aging, the development effect does not decline significantly, which further indicates that the material can adequately adsorb to the latent fingerprint residue and indirectly supports the positive role of its hygroscopicity in fingerprint development.

#### 3.2.4. Stability Test of Fingerprints Developed by Cs_2_NaBi_0.6_Er_0.4_Cl_6_

**Figure 13:**
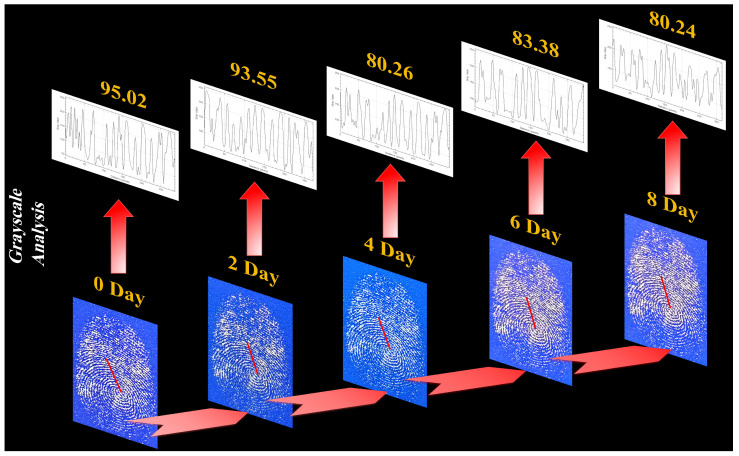
Representative images of latent fingerprints developed with Cs_2_NaBi_0.6_Er_0.4_Cl_6_ nanoparticles after different post-development storage times.

**Figure 14:**
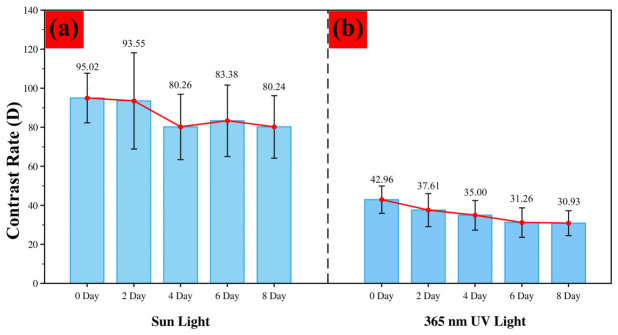
(**a**) Contrast (*D*) of latent fingerprints at different time intervals after development with the Cs_2_NaBi_0.6_Er_0.4_Cl_6_ nanoparticle material under natural illumination; (**b**) contrast (*D*) of latent fingerprints developed with Cs_2_NaBi_0.6_Er_0.4_Cl_6_ nanoparticles at different post-development storage times under 365 nm (UV) illumination.

To evaluate the post-development stability of fingerprints developed with the Cs_2_NaBi_0.6_Er_0.4_Cl_6_ double-perovskite nanomaterial, latent fingerprints were prepared on frosted plastic substrates, developed by dusting, and then stored under general environmental conditions. Photographs were taken at 0, 2, 4, 6, and 8 days after development under natural light and 365 nm UV light, respectively. The contrast was subsequently calculated according to the method described in Section 2; the results are shown in Figure 13 and Figure 14. The data show that with increasing storage time, the contrast of the developed fingerprints under white light decreases slightly, but the fingerprint ridges remain clearly discernible; under UV excitation, the decrease in fluorescence contrast is even more moderate. Specifically, under natural illumination, the contrast *D* of the as-developed fingerprint is 95.02 at 0 days and decreases to 80.24 at 8 days, a reduction of 14.78; under 365 nm UV light, the contrast *D* is 42.96 at 0 days and 30.93 at 8 days, a reduction of only 12.03 in Table 5. These results indicate that the fluorescent development nanopowder prepared in this work possesses good stability over relatively long periods and that its fluorescence performance remains fairly stable under ambient conditions, maintaining a reliable ability to develop fingerprints.

## 4. Conclusions

In this study, Cs_2_NaBi_0.6_Er_0.4_Cl_6_ double-perovskite nanoparticles were synthesized by a solvothermal method and used as a fingerprint development powder for latent fingerprint visualization on rough non-porous substrates. The prepared material exhibited relatively uniform particle morphology, good dispersibility, possible intrinsic moisture affinity, and orange-red emission under 365 nm UV excitation.

Comparative experiments showed that, compared with conventional powders, the prepared powder produced improved fingerprint visualization on frosted plastic substrates. The *D* values for natural-residue fingerprints and oil-contaminated model latent fingerprints reached 51.21 and 35.87, respectively. In addition, the material maintained satisfactory visualization capability for aged fingerprints and acceptable post-development stability during extended storage.

More importantly, the results indicate that the excellent development performance of the prepared powder mainly originates from selective adhesion assisted by moisture affinity rather than from fluorescence intensity alone. The moisture-affinitive nature of the particles may facilitate water-assisted capillary interactions with fingerprint residues, enhancing preferential retention in ridge regions and suppressing background contamination on rough substrates.

These findings provide a physical understanding of particle–residue interactions at rough, complex interfaces and offer a useful strategy for designing functional fingerprint development powders for difficult non-porous surfaces.

## Figures and Tables

**Table 1:** Substrates and fingerprint development powders used for fingerprint development.

		Glass Slide	Stainless-Steel Cup	Compact Disc	Smooth Plastic Cup	Frosted Plastic Cup
Sweat fingerprints	Gold powders	A1	A2	A3	A4	A5
	Silver powders	B1	B2	B3	B4	B5
	Red fluorescent powders	C1	C2	C3	C4	C5
	Cs_2_NaBi_0.6_Er_0.4_Cl_6_ Nanoparticle powders	D1	D2	D3	D4	D5
Oil fingerprints	Gold powders	a1	a2	a3	a4	a5
	Silver powders	b1	b2	b3	b4	b5
	Red fluorescent powders	c1	c2	c3	c4	c5
	Cs_2_NaBi_0.6_Er_0.4_Cl_6_ Nanoparticles powders	d1	d2	d3	d4	d5

**Table 2:** TRPL (time-resolved photoluminescence) decay lifetime fitting results of Cs_2_NaBi_0.6_Er_0.4_Cl_6_.

	$A_{1}$	$\tau_{1}$ (μs)	$A_{2}$	$\tau_{2}$ (μs)	$A_{3}$	$\tau_{3}$ (μs)	$\tau_{\mathrm{ave}}$ (μs)
Cs_2_NaBi_0.6_Er_0.4_Cl_6_	36,470.92	203.49	135,356.59	2004.70	4373.23	4037.38	2080.68

**Table 3:** Contrast quantified as the difference in intensity between valleys and ridges (*D*), as the ratio of intensity in valleys to ridges (*R*) and as the Michelson contrast ($C_{m}$).

		*D*|Error Bar (%)	*R*|Error Bar (%)	$C_{m}$|Error Bar (%)
Sweat fingerprints	Gold powders	35.67|4.30	0.77|19.74	0.13| 25.11
	Silver powders	14.23|6.25	0.89|10.25	0.06|18.64
	Red fluorescent powders	10.97|5.49	0.94|5.44	0.03|7.71
	Cs_2_NaBi_0.6_Er_0.4_Cl_6_ nanoparticle powders	51.21|4.85	0.72|7.94	0.17|21.46
Oil fingerprints	Gold powders	16.73|5.11	0.88|13.46	0.06|4.82
	Silver powders	21.86|4.64	0.81|3.87	0.10|17.29
	Red fluorescent powders	11.06|6.36	0.93|9.98	0.04|13.6
	Cs_2_NaBi_0.6_Er_0.4_Cl_6_ Nanoparticle powders	35.87|4.55	0.82|14.91	0.10|24.48

**Table 4:** Contrast (*D*) of aged latent fingerprints developed by Cs_2_NaBi_0.6_Er_0.4_Cl_6_ nanoparticles under natural light and 365 nm UV light.

	White Light|Error Bar (%)	365 nm UV Light|Error Bar (%)
0 days	95.02|11.21	42.96|5.96
2 days	93.82|19.21	39.78|8.56
4 days	97.97|19.94	39.15|8.62
6 days	83.03|18.20	31.57|7.24
8 days	83.72|14.86	40.61|7.70
10 days	106.03|25.96	46.65|9.83

**Table 5:** Contrast (*D*) of latent fingerprints at different time intervals after development with Cs_2_NaBi_0.6_Er_0.4_Cl_6_ nanoparticles under natural light and 365 nm UV light.

	White Light|Error Bar (%)	365 nm UV Light|Error Bar (%)
0 days	95.02|12.75	42.96|6.97
2 days	93.55|24.68	37.61|8.45
4 days	80.26|16.76	35.00|7.58
6 days	83.38|18.32	31.26|7.56
8 days	80.24|19.94	30.93|6.41

## Data Availability

The data presented in this article and the code used in this study are available from the corresponding author upon request.
